# Directions for Using Roberts’ Vulcanizing Apparatus

**Published:** 1859-11

**Authors:** 


### DIRECTIONS FOR USING ROBERTS' VULCANIZING APPARATUS.

This apparatus being the most simple of any now in use for vulcanizing rubber or gutta percha, is more easily managed than those more complicated; yet some little direction is required to enable a beginner to use it with facility. And first, the water chamber should be almost filled with water, at the beginning, then the plates put in, and the work put in on these. Then the top put on and screwed down as tightly as possible, taking care that the packing is good, and properly adjusted. The taps should be screwed down as firmly as possible with an ordinary wrench.

The fire may now be started, and the steam raised in the heater, till the thermometer rises to about 260° within th first half hour, then raise to 290°, during the next half hour, then raise it up to 305° during the next two hours; this completes the operation for the common gum.

For the fine, or recently improved gum, raise the thermometer to 250° during the first hour, then to 290° during the second, and to 320° during the third hour, then to 340° during the fourth, then retain it at 345° during the fifth and sixth hours, which completes the work.

At the expiration of this time, the steam should be let off by the escape valve, the fire put out, top taken off, and the pieces removed. These are cooled down gradually, and the pieces removed from the moulds and finished up.

